# Editorial: Physical fitness via advanced technology—ICT solutions for healthier ageing

**DOI:** 10.3389/fspor.2024.1484154

**Published:** 2024-09-17

**Authors:** Sonja Jungreitmayr, Verena Venek

**Affiliations:** ^1^ Department Sport and Exercise Science, University of Salzburg, Salzburg, Austria; ^2^ Lakeside Labs GmbH, Klagenfurt, Austria

**Keywords:** physical activity, healthy ageing, ICT, physical fitness, technology—assistive/supportive

**Editorial on the Research Topic**
Physical fitness via advanced technology—ICT solutions for healthier ageing

## Introduction

The use of information and communication technology (ICT) is increasingly pivotal in addressing the health challenges posed by an aging global population. This Research Topic, “*Physical fitness via advanced technology—ICT solutions for healthier ageing*,” showcases approaches leveraging these technologies to enhance physical fitness and health among the elderly. The following articles illuminate diverse facets of this integration, providing insights into how advanced technology can promote healthier aging.

### From accelerometers…

Our research topic begins with the tools that have been used to monitor activity since the start of this millennium. Berger et al. present a scoping review of the measurement of physical activity via accelerometers. Since they are relatively small devices, accelerometers can be worn in many positions while recording a variety of markers, such as physical activity per minute, intensity of activity, as well as type of activity, e.g., postural components of physical activity. Lack of usable data, technical errors of the device and a disregard for the wearing protocol are cited as challenges when using such technologies. However, the data show that the latter was not reported when devices were worn at the wrist, i.e., built into smartwatches or similar wearables. Furthermore, the article discusses how the combination of accelerometers with digital applications could offer promising benefits in promoting physical activity.

### …to smartwatches…

One article of this topic Düking et al. discusses the utility of smartwatches in promotion of physical activity by improving the accuracy of daily step counts. It shows how these devices not only serve as monitoring tools, but also enhance body awareness, especially in people who initially underestimate their physical activity. By promoting better self-awareness, smartwatches encourage users to engage in more consistent physical activity, which contributes to better health outcomes. The results suggest a dual benefit of wearable technology: more accurate activity tracking and increased user motivation through enhanced body awareness.

### …the use of and experience with digital technology

The findings of the next article may be of great interest to patients in cardiovascular disease rehabilitation. This patient group was researched by Zeller et al. regarding their experiences with and usage of digital technology. Notably participants stated to predominantly use smartwatch and/or smartphone features. Besides step counting, other markers such as heart-rate and blood-pressure stand out to be well controlled via modern means. However, it is found that a quite substantial number of patients expressed negative attitudes towards the broader use of digital technologies, be it out of past experiences, low affinity in general, or lack of knowledge on usage, hence dependency in those areas. Simplicity, convenience, and accessibility, as stated by the authors, are key factors when it comes to using digital tools in rehabilitation.

### …is constantly thriving, now also engaging AR and other means

Those findings are in line with Desai et al., who examine different mixed reality prompts for people with dementia. This article highlights the need for a better understanding of cueing modalities for this group with special needs. With optimized modalities, they come one step closer to improving their quality of life, as autonomous living can be promoted through assistive tools. It has been found that different data presentation modes, such as graphs and numerical data, are less perceived when prompted than simple shapes or, even better, the human voice. Intuitive design and audible feedback on actions performed seem to be essential in the design of helpful tools. With the help of AI, this feedback can adapt to the current situation and thus provide useful information exactly when it is needed. Moreover, communication with the system could be facilitated by gestures that are prompted by the system and responded to by the users, according to the article. However, the technology of these systems still needs to be fully developed if they are to be well received.

### Proper integration of technology will be essential in the future of ICT in ageing

The articles in this Research Topic collectively highlight several aspects in utilizing ICT for healthier aging. Nevertheless, the advancements in digital technology do not stop at smartwatches, smartphones and prompting applications, but also include wearable technology, personalized fitness programs, fall prevention devices, and disease management systems underscoring the transformative potential of modern means. As research continues to evolve, it is crucial to address challenges such as data privacy, user accessibility, and technology adoption among older adults. Ensuring that these technologies are user-friendly and accessible will be key to their successful implementation and widespread adoption.

The integration of ICT in promoting physical fitness and health among the elderly is a promising field with far-reaching implications. By leveraging the capabilities of ICT in combination with other advancing technologies, such as Artificial Intelligence, we can create adaptive and personalized solutions that meet the unique needs of the aging population. This holistic approach not only addresses the physical aspects of aging but also enhances mental and emotional well-being, promoting a healthier and more fulfilling life for older adults. As we move forward, the collaboration between technology developers, healthcare providers, and researchers will be crucial in realizing the full potential of these advancements, ensuring that the benefits of ICT are accessible to all, regardless of age or technological proficiency.

